# Two-step hierarchical neural network for classification of dry age-related macular degeneration using optical coherence tomography images

**DOI:** 10.3389/fmed.2023.1221453

**Published:** 2023-07-19

**Authors:** Min Hu, Bin Wu, Di Lu, Jing Xie, Yiqiang Chen, Zhikuan Yang, Weiwei Dai

**Affiliations:** ^1^Changsha Aier Eye Hospital, Changsha, China; ^2^Department of Retina, Shenyang Aier Excellence Eye Hospital, Shenyang, China; ^3^Department of Retina, Shenyang Aier Optometry Hospital, Shenyang, China; ^4^Institute of Computing Technology, Chinese Academy of Sciences, Beijing, China; ^5^Aier Institute of Optometry and Vision Science, Changsha, China; ^6^Anhui Aier Eye Hospital, Anhui Medical University, Hefei, China

**Keywords:** optical coherence tomography (OCT), age-related macular degeneration (AMD), nascent geographic atrophy (nGA), convolutional neural network (CNN), deep learning

## Abstract

**Purpose:**

The aim of this study is to apply deep learning techniques for the development and validation of a system that categorizes various phases of dry age-related macular degeneration (AMD), including nascent geographic atrophy (nGA), through the analysis of optical coherence tomography (OCT) images.

**Methods:**

A total of 3,401 OCT macular images obtained from 338 patients admitted to Shenyang Aier Eye Hospital in 2019–2021 were collected for the development of the classification model. We adopted a convolutional neural network (CNN) model and introduced hierarchical structure along with image enhancement techniques to train a two-step CNN model to detect and classify normal and three phases of dry AMD: atrophy-associated drusen regression, nGA, and geographic atrophy (GA). Five-fold cross-validation was used to evaluate the performance of the multi-label classification model.

**Results:**

Experimental results obtained from five-fold cross-validation with different dry AMD classification models show that the proposed two-step hierarchical model with image enhancement achieves the best classification performance, with a f1-score of 91.32% and a kappa coefficients of 96.09% compared to the state-of-the-art models. The results obtained from the ablation study demonstrate that the proposed method not only improves accuracy across all categories in comparison to a traditional flat CNN model, but also substantially enhances the classification performance of nGA, with an improvement from 66.79 to 81.65%.

**Conclusion:**

This study introduces a novel two-step hierarchical deep learning approach in categorizing dry AMD progression phases, and demonstrates its efficacy. The high classification performance suggests its potential for guiding individualized treatment plans for patients with macular degeneration.

## 1. Introduction

Age-related macular degeneration (AMD) (1) is an ocular disease that manifests with a degenerative change in the retina and choroid of the macular region. According to the World Health Organization, ~1.3 billion people globally suffer from varying degrees of vision loss, with AMD as the third leading cause of vision loss among patients (2, 3). AMD predominantly affects individuals over the age of 50, and its prevalence increases with advancing age (4). As the global population continues to age, the number of people affected by AMD is expected to rise, further highlighting the importance of understanding and addressing this debilitating ocular disease (5).

Two distinct categories of AMD are typically distinguished considering both clinical and pathological features: the atrophic or dry form, and the exudative or wet form. The exudative form is defined by the presence of abnormal retinal changes caused by the growth of newly formed vessels within the macula. The dry form, on the other hand, is characterized by a progressive course that culminates in degeneration of the retinal pigment epithelium (RPE), thickening of the Bruch membrane, and photoreceptor loss (6). In its late stages, dry AMD manifests with localized RPE degeneration and photoreceptor loss, known as geographic atrophy (GA), which ultimately leads to progressive and irreversible loss of visual function.

Early detection and intervention of GA can facilitate the timely intervention from clinicians and better management of the disease, leading to improved patient outcomes. The recent approval by the U.S. Food and Drug Administration of a new treatment for GA accentuates the importance of early detection of this condition (7, 8). The identification of GA is also valuable in understanding the natural progression of AMD and characterizing the disease spectrum. However, GA is typically small in its nascent stage and can be difficult to identify accurately, even for experienced clinicians. This difficulty is exacerbated by the fact that the imaging characteristics of GA are heterogeneous in shape, size, and location, making it challenging to detect and differentiate from other kind of retinal lesions such as drusen. By accurately detecting the earliest signs of GA, clinicians can provide timely and effective interventions, leading to better management of the condition and improved patient outcomes.

**Figure 1:**
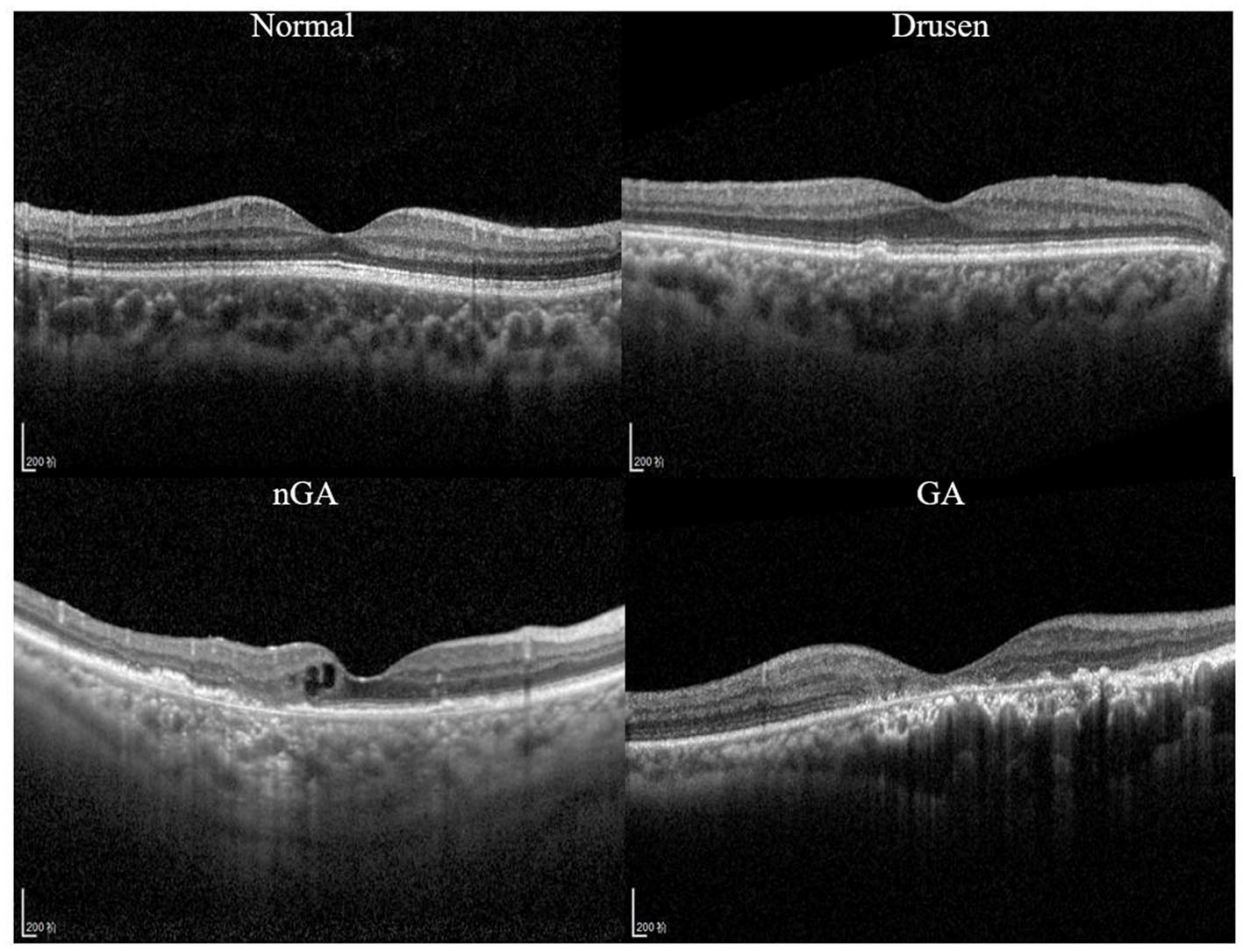
Optical coherence tomography (OCT) images of a healthy retina, drusen, nascent geographic atrophy (nGA) and geographic atrophy (GA).

A recent study (9) demonstrated that *nascent GA* (nGA) is a strong predictor for the development of GA, providing supportive evidence of the potential value of nGA as a surrogate endpoint in future intervention trials for the early stages of AMD. In OCT images, the presence of nGA is defined as the presence of the subsidence of the inner nuclear layer (INL) and outer plexiform layer (OPL), and/or the presence of a hyporeflective wedge-shaped band (Figure 1).

OCT has emerged as a critical imaging technique for the diagnosis and classification of atrophy, with a consensus established based on the assessment of OCT images (10). The Classification of Atrophy Meetings (CAM) group has promulgated guidelines for the employment of OCT in the diagnosis of dry AMD. According to CAM's consensus, nGA was suggested to be retained as the term to describe incomplete retinal pigment epithelium and outer retinal atrophy (iRORA) in the absence of choroidal neovascularization (CNV) (10). To ensure accurate diagnosis and treatment, reproducibility is crucial in identifying iRORA and nGA with OCT.

Advances in OCT technology and the integration of artificial intelligence algorithms will likely improve the accuracy and reliability of iRORA identification as clinicians and reading centers become more familiar with OCT findings (11). Recently numerous studies have reported the application of deep convolutional neural networks (CNNs) in the diagnosis of AMD (12–15). In these studies, promising results have been demonstrated in detecting AMD with medical images such as color fundus photography and OCT. With the aid of deep learning algorithms and medical image analysis, identifying the early signs of GA (i.e., nGA) has become increasingly feasible.

However, no previous studies have explored the feasibility of detecting the early stages of dry AMD. One of the challenges is that currently available datasets for AMD classification tend to focus on the more advanced phases of the disease, and do not contain sufficient examples of early stages. The variability in image quality, diagnosis, and definition of AMD phases across different datasets further complicates the task of detecting early stages of the disease using CNNs.

In seeking to improve the early detection of dry AMD, including the characteristics such as nGA, we developed and validated a novel two-step hierarchical CNN model along with image enhancement for dry AMD classification. The major contributions of this research can be outlined as follows:

- To the best of our knowledge, this is the first investigation to use CNNs to classify the early stages of dry AMD, including nGA.
- To leverage the domain expertise regarding the characteristics of OCT images, including those of nGA, we combine image enhancement and hierarchical classification techniques, effectively highlighting features associated with nGA while preserving the overall integrity of the OCT images.
- The proposed method demonstrates promising classification results and can be utilized as a useful computer-aided diagnostic tool for clinical OCT-based AMD diagnosis.

## 2. Materials and methods

### 2.1. Datasets and labeling

OCT images in this study were collected from dry AMD patients admitted to the Shenyang Aier Excellence Eye Hospital (Shenyang, China) in 2019–2021. The device used to obtain the images was a Heidelberg Spectralis HRA + OCT (Heidelberg, Germany), and the scan length was 6 mm × 6 mm. A total of 3,401 qualified OCT images from 338 patients were selected for model development. Each image was classified by a retinal specialist with over 15 years of clinical expertise. Example OCT images of both healthy retinas and dry AMD classes are presented in Figure 1. These images were divided into a training dataset (~80% of the patients) for model development and a validation dataset (~20% of the patients) for validating the models based on the patient's identification number. The partitioning of the dataset into approximate proportions of 80 and 20% is contingent upon the distribution of patients rather than images. Given that each patient may present an unequal number of OCT images acquired during a single visit, the precise ratios of the division may exhibit minor discrepancies across the five-fold cross validation dataset.

The dataset, as detailed in Table 1, contains 712 images of normal, 1,167 images of drusen, 711 images of nGA, and 811 images of GA. We conducted five-fold cross-validation to evaluate the model performance, which involved partitioning the dataset into five equally sized folds, as described in Table 1. In each iteration of the five-fold cross-validation, four of the five subsets are used for training the model, while the remaining subset is utilized for testing its performance. This process is carried out five times, ensuring that each subset serves as the test set once. The final performance metric is obtained by computing the average of the results from each of the five iterations. This approach allows for a more accurate assessment of the model's generalizability and performance on diverse subsets of the data, reducing the risk of overfitting (16).

**Table 1:** Details of the training and validation datasets in five-fold cross validation.

	**Fold 1**		**Fold 2**		**Fold 3**		**Fold 4**		**Fold 5**	
	**Train**	**Val**	**Train**	**Val**	**Train**	**Val**	**Train**	**Val**	**Train**	**Val**
No of patients (%)	260 (79.5)	67 (20.5)	260 (79.5)	67 (20.5)	261 (79.8)	66 (20.2)	263 (79.9)	66 (20.1)	266 (80.9)	63 (19.1)
No of images (3,401)	2,720	681	2,806	595	2,587	814	2,742	659	2,749	652
Normal (712)	591	121	621	91	501	211	566	146	569	143
Drusen (1,167)	970	197	875	292	937	230	941	226	945	222
nGA (711)	554	157	592	119	564	147	560	151	574	137
GA (811)	605	206	718	93	585	226	675	136	661	150
Total images (patients)	3,401 (338)									

### 2.2. Development of a deep learning classifier

#### 2.2.1. Image preprocessing

**Figure 2:**
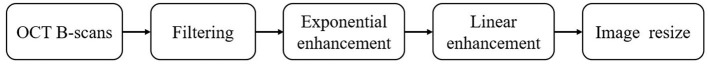
Flowchart of image enhancement methods adopted in preprocessing.

**Figure 3:**
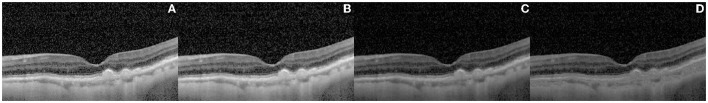
Results of the image enhancement procedure on a representative OCT image of retinal layers. The original image is shown in **(A)**, while **(B–D)** illustrate the effect of different enhancement techniques, namely anisotropic diffusion filtering **(B)**, exponential enhancement **(C)**, and linear enhancement **(D)**, on the image. The final image, shown in panel **(D)**, highlights the retinal layer by adapting the pixel values within a specific intensity range, making it more distinguishable from other features in the image.

Image enhancement was performed to improve the quality and contrast of the OCT images, as they are inevitably susceptible to speckle noise, detection noise, and photon shot noise (17–19). Numerous studies have investigated the impact of image enhancement techniques on OCT images (20–22). The current study applied filtering, exponential enhancement, and linear enhancement to enhance the visibility of important features in the images, such as drusen and atrophic areas, as shown in Figures 2, 3. This process was intended to improve the performance of the CNN models by providing them with clear and informative images.

The outcome of the image enhancement procedure, performed on a representative image, is presented in Figure 3. The original image is presented in Figure 3A. Prior to further processing, an anisotropic diffusion filtering method (23) was employed to eliminate the speckle noise and accentuate the contrast between the distinct retinal layers, as demonstrated in Figure 3B. In the process of anisotropic diffusion, the parameters were set as alpha = 0.05 and K = 0.3. Subsequently, exponential enhancement (24) was applied to account for the light attenuation in OCT images and improve the image contrast, leading to the image of Figure 3C. The exponential enhancement was performed with parameters c = 0.4 and gamma = 1.1. Following this, a linear enhancement (25) method was implemented to further emphasize the layer by adapting the pixel values within a specific intensity range, thus rendering the layer more apparent and distinguishable from other features present in the image. The linear enhancement was applied with coefficient m = 20. The final result of image enhancement is demonstrated in Figure 3D.

#### 2.2.2. Hierarchical classification

**Figure 4:**
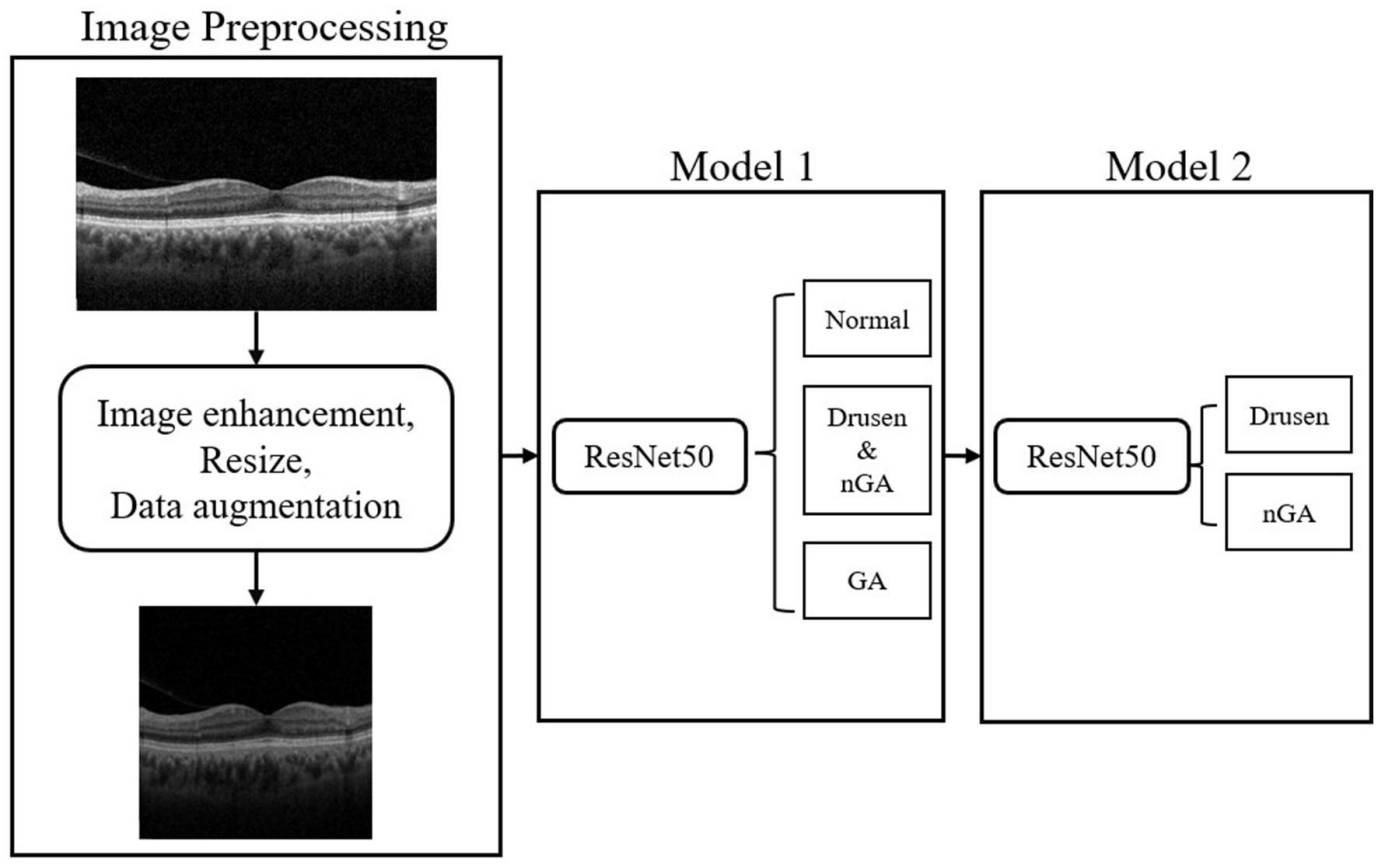
Flowchart of our proposed hierarchical classification model.

Within three phases of dry AMD, drusen-associated atrophy and nGA are both types of age-related macular degeneration that share similar imaging characteristics, making them difficult to differentiate in OCT images. To tackle this problem, hierarchical models were developed to conduct a two-step classification of dry AMD. Hierarchical models perform better than flat models in image classification (26). Recent studies have leveraged the hierarchical organization of object categories to break down classification tasks into multiple stages, leading to successful outcomes (27–29). In this research, we implemented hierarchical classification by training two models, as depicted in the flowchart presented in Figure 4. The initial model generates a general dry AMD classification, distinguishing between normal, early stages of GA (i.e., drusen or nGA), and GA. The subsequent model concentrates on discriminating between drusen and nGA. Upon merging the outputs of the two models, a final classification result consisting of four labels is obtained.

In the hierarchical classification method, the base models serve as the primary feature extractor and the extracted features are then used to classify the images into their respective classes at different levels of the hierarchy. CNNs, which exhibit advantages in many application areas including medical diagnosis (30–33), are selected as the candidates of the base model in the hierarchical classification. Four CNNs, specifically Normalizer-Free ResNet-50 (34), Xception (35), DenseNet169 (36), and EfficientNetV2 (37), were evaluated as classification models for dry AMD. These models are widely recognized for their remarkable performance in a variety of computer vision and medical image analysis applications, making them suitable for this study. All the models were initially trained on a vast labeled dataset known as ImageNet (38) and were subsequently fine-tuned on OCT images. The final layer of these models was removed, and a new fully connected layer with an output size of four was inserted to represent the four distinct classes (normal, drusen, nGA, and GA) for classification. To minimize the computation cost and select the model with the best performance for further analysis, we conducted a hold-out validation using only the Fold 1 dataset of Table 1, rather than employing five-fold cross-validation, to compare different CNN models.

We trained the deep learning models using PyTorch (39), a commonly used library in the deep learning community. During the training process, we updated the model parameters using the Adam optimizer (learning rate of 0.00002) for every minibatch of four images. The training was stopped after 20 epochs once the accuracy values no longer increased or started to decrease. All experiments were conducted on a server with Intel Core i5-10600KF, using an NVIDIA GeForce RTX 3090 24GB GPU for training and validation, with 32GB RAM.

Cross-entropy loss curves were plotted against the number of training steps to visualize the model convergence during training. The loss function estimates the discrepancy between the predicted and actual labels and is employed to optimize the model during training. The loss curves enable us to observe how the models learn and converge toward the optimal solution.

Gradient-weighted class activation maps (Grad-CAM) was also employed to generate heatmaps that provide visual explanations of the classification model's predictions by highlighting the areas in retinal images that contributed significantly to the classification of normal, drusen, nGA, and GA cases. Grad-CAM is a visualization technique that employs the gradients of target class scores with respect to feature maps in the final convolutional layer to produce a coarse localization map, highlighting the important regions in the input image for a specific class prediction (40). This visualization technique not only assists in the interpretation of the model's decisions but also aids in identifying potential misclassifications or biases, consequently improving the model's usability and clinical applicability.

#### 2.2.3. Evaluation metrics

To assess the efficacy of the four models, various metrics were employed, including accuracy, sensitivity, specificity, and the f1-score for each class, as well as the macro-f1 and kappa coefficients for overall classification performance. Sensitivity gauges the ratio of true positive predictions for a given class, while specificity measures the proportion of true negative predictions. The f1-score is the harmonic mean of precision and recall, and offers a holistic measure of the model's accuracy for a particular class. The macro-f1 and kappa coefficients were used to evaluate the models' overall performance. The macro-f1 score is the arithmetic mean of f1-scores for each class, while the kappa coefficient measures the level of agreement between the predicted and actual labels, considering the possibility of chance agreement.

## 3. Results

### 3.1. Base model selection

Table 2 presents the results given by the four CNN models evaluated on a single fold of the validation dataset, with each of the four rows corresponding to the classification metrics for individual classes, i.e., normal, drusen, nGA, and GA.

**Table 2:** Classification results (percentage) of four CNN models on one fold of validation dataset.

**Methods**	**Classes**	**Accuracy**	**Sensitivity**	**Specificity**	**F1**	**Macro-f1**	**Kappa**
EfficientNetV2	Normal	98.66	100.00	98.41	95.79	89.93	94.01
	Drusen	94.96	92.12	97.69	94.72		
	nGA	92.44	89.08	93.28	82.49		
	GA	96.13	81.72	98.80	86.86		
DenseNet169	Normal	99.33	100.00	99.21	97.85	90.35	94.33
	Drusen	95.46	94.18	96.70	95.32		
	nGA	92.61	88.24	93.70	82.68		
	GA	95.80	79.57	98.80	85.55		
Xception	Normal	98.82	100.00	98.61	96.30	88.12	93.88
	Drusen	95.29	91.78	98.68	95.04		
	nGA	91.09	91.60	90.97	80.44		
	GA	94.62	72.04	98.80	80.72		
ResNet50NF	Normal	99.66	100.00	99.60	98.91	**92.08**	**95.45**
	Drusen	94.96	90.07	99.67	94.60		
	nGA	92.61	94.12	92.23	83.58		
	GA	97.31	89.25	98.80	91.21		

Each rows gives the classification metrics for individual classes: normal, drusen, nGA and GA. The best results are shown in bold.

Notably, all four CNN models produce commendable results for the normal, drusen, and GA categories, with all f1 scores exceeding 90%. However, the classification performance for nGA is generally inferior, with the EfficientNetV2, DenseNet169, Xception, and ResNet50NF models giving f1 scores of 82.49%, 82.68%, 80.44%, and 83.58%, respectively. Among the evaluated models, the ResNet50NF model produces the highest overall f1 scores, with 98.91% for normal, 94.60% for drusen, 83.58% for nGA, and 91.21% for GA. The macro-f1 and kappa scores using ResNet50NF are 92.08% and 95.45%, respectively.

**Figure 5:**
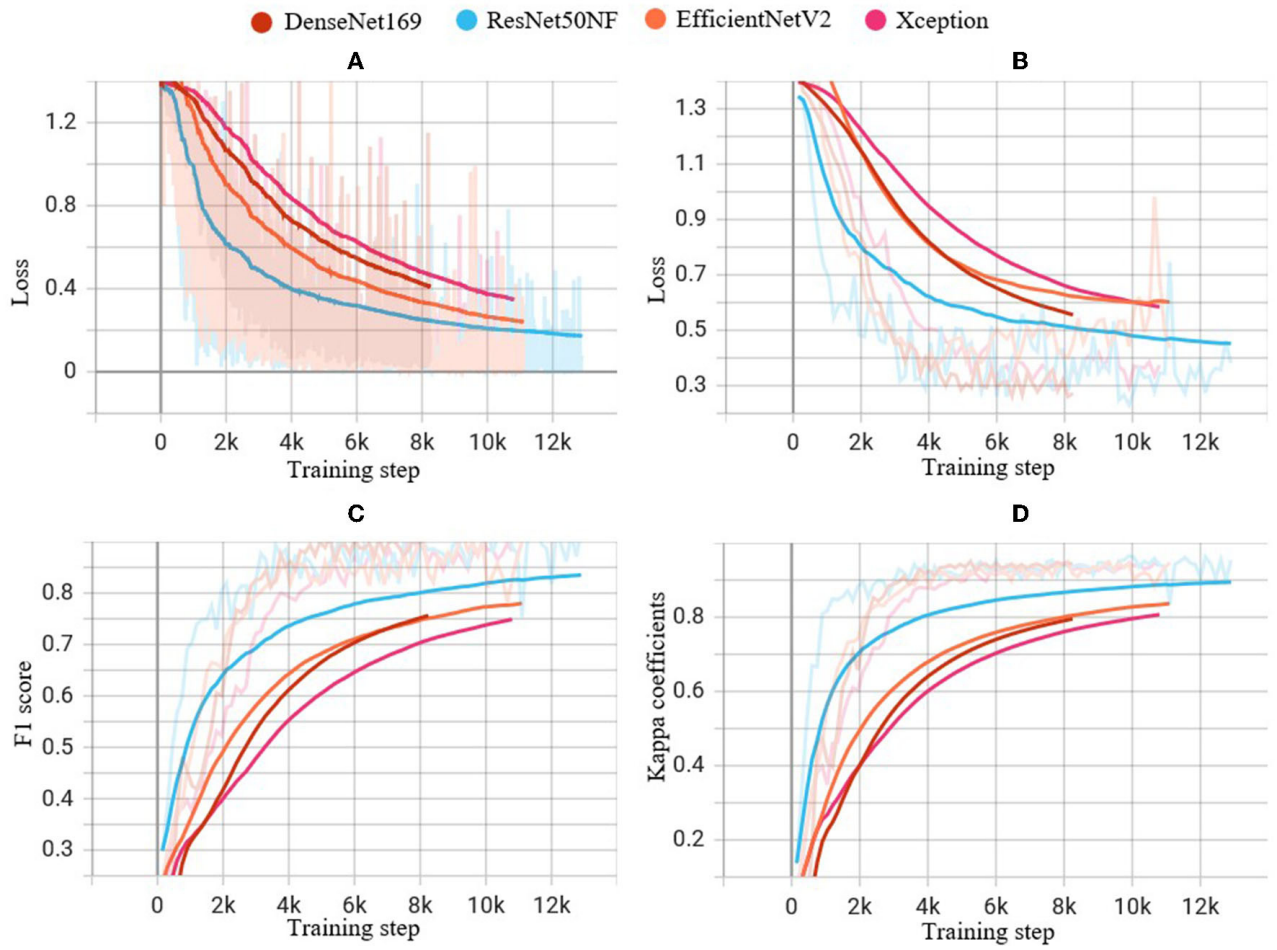
Performance of experiments on the four CNN models during the training steps: **(A)** cross-entropy loss on the training dataset, **(B)** cross-entropy loss on the validation dataset, **(C)** f1 score on the validation dataset, and **(D)** kappa coefficients on the validation dataset. The light-colored lines indicate the actual values of each metric at each step of the training process, while the solid lines indicate smoothed curves generated from the actual values for improved visual clarity.

Figure 5 illustrates the performance of the four CNN models on the training dataset in terms of cross-entropy loss (A), and on the validation dataset in terms of cross-entropy loss (B), macro-f1 score (C), and kappa coefficient (D) during the training process. Notably, ResNet50NF achieves the highest macro-f1 score and kappa coefficient, while also exhibiting fastest convergence, as evidenced in Figures 5A, B. Given the superior classification performance and fast convergence of ResNet50NF, this CNN was selected as the base model for the subsequent hierarchical classification. The proposed model with image enhancement and hierarchical classification achieves macro-f1 of 91.32 ± 9.06%, and kappa score of 96.09 ± 4.44%.

### 3.2. Ablation study

We compared the performance of each components of the proposed method by ablation study. The ablation results are presented in Table 3. The accuracy, sensitivity, specificity, and f1 score are listed for the normal, drusen, nGA, and GA classes. The macro-f1 and kappa values are also displayed as metrics of the overall classification performance.

**Table 3:** Ablation study results (%) of the baseline model, the model with only image enhancement, the model with only hierarchical classification, and the proposed method.

**Methods**	**Classes**	**Accuracy**	**Sensitivity**	**Specificity**	**F1**	**Macro-f1**	**Kappa**
ResNet50NF(baseline)	Normal	98.99 ± 1.44	99.73 ± 0.76	98.78 ± 1.65	97.75 ± 3.02	84.96 ± 3.98	93.01 ± 2.19
	Drusen	90.33 ± 4.41	86.11 ± 11.41	92.05 ± 6.8	85.24 ± 7.97		
	nGA	86.16 ± 4.53	67.82 ± 19.39	91.03 ± 6.0	66.79 ± 11.17		
	GA	95.55 ± 2.95	86.09 ± 6.89	98.34 ± 1.78	90.07 ± 5.42		
Baseline+ Imageenhancement	Normal	98.74 ± 0.72	98.27 ± 3.6	98.86 ± 1.06	97.06 ± 1.57	85.17 ± 4.58	93.23 ± 1.67
	Drusen	89.89 ± 4.79	86.18 ± 13.2	91.37 ± 8.05	84.68 ± 8.83		
	nGA	86.78 ± 4.76	68.31 ± 20.94	91.69 ± 6.15	67.83 ± 12.18		
	GA	95.94 ± 3.36	87.78 ± 7.97	98.39 ± 1.96	91.11 ± 6.52		
Baseline+ Hierarchicalclassification	Normal	99.48 ± 0.87	99.45 ± 1.15	99.54 ± 0.92	99.01 ± 1.76	89.78 ± 4.91	95.46 ± 2.35
	Drusen	93.26 ± 5.98	89.34 ± 13.98	94.75 ± 7.25	88.86 ± 8.77		
	nGA	89.96 ± 5.13	73.24 ± 20.38	93.85 ± 6.53	72.74 ± 12.88		
	GA	96.84 ± 2.97	91.57 ± 7.89	99.07 ± 1.68	93.28 ± 6.49		
Proposed method	Normal	99.68 ± 0.9	99.67 ± 0.92	99.68 ± 0.89	99.12 ± 2.45	**91.32** **±9.06**	**96.09** **±4.44**
	Drusen	94.12 ± 6.63	90.04 ± 17.64	95.89 ± 5.16	90.3 ± 13.23		
	nGA	91.56 ± 9.26	81.87 ± 13.4	94.0 ± 10.76	81.65 ± 14.65		
	GA	97.12 ± 3.95	93.7 ± 13.29	98.57 ± 1.92	94.22 ± 7.05		

The best results are shown in bold.

The baseline model achieves a high f1 score on the normal (98.99%) and GA (90.07%) classes, but has a limited ability to classify drusen (85.24%) and nGA (66.79%) classes. Image enhancement slightly improves the classification performance of nGA (from 66.79% to 67.83%) and GA (from 90.07% to 91.11%), but does not improve the performance over the baseline for the normal and drusen classes. By applying hierarchical classification to the baseline model, the classification performance of nGA was improved (from 66.79% to 72.74%, and GA from 90.07% to 93.28%). The proposed method which includes image enhancement along with hierarchical structure, achieves the highest accuracy for all classes, with a significant improvement on the nGA class (from 66.79% to 81.65%).

In terms of sensitivity and specificity, the proposed method outperforms the other two models in all classes, indicating that it can better distinguish between true positives and true negatives for each class. The macro-f1 and kappa coefficient for the proposed method, are 91.32% and 96.09% respectively, which are higher than the corresponding values without image enhancement and hierarchical structure. This demonstrates that the proposed model achieves better overall performance.

### 3.3. Model explainability

The heatmaps generated through Gram-CAM confirm that the proposed model produces an accurate diagnosis by leveraging distinctive features and pertinent regions or lesions within the image. As illustrated in Figure 7, the heatmaps demonstrate that the proposed model diagnoses the pathology based on the correct lesion. In the validation dataset, the model successfully detected pathological changes and identified distinguishing features for the three representative OCT results of the eyes that developed drusen, nGA, and GA. In particular, as depicted in the nGA example, the proposed model correctly highlighted the area where subsidence of OPL and INL were observed (gray mark in Figure 7C).

## 4. Discussion

The current study aimed to investigate the performance of classifying early stages of dry AMD based on OCT images. The study proposed a novel hierarchical classification method that combines image enhancement with the ResNet50NF base CNN, which improves the classification performance of nGA, a challenging task for both ophthalmologists and deep learning models.

While most related studies have predominantly focused on using OCT images to classify exudative AMD, particularly those involving CNV, there has been a noticeable lack of research on dry AMD stages such as GA. These investigations (41, 42) have demonstrated promising results in identifying CNV within OCT images. However, when comparing CNV characteristics with those of dry AMD stages such as GA, several key differences in image features can be observed. For example, CNV typically presents with subretinal fluid, hemorrhages, and a distinct network of new blood vessels, whereas GA is characterized by a more uniform thinning of the retinal pigment epithelium and photoreceptor layers, along with the absence of fluid or hemorrhages.

GA is a chronic ocular condition that causes a decline in visual function, leading to difficulties in performing everyday activities such as reading, recognizing faces, and driving, ultimately resulting in a loss of independence. As the disease progresses and the lesions expand, patients often experience a slow and steady decline in visual function. Managing and treating GA can be particularly challenging because the available treatment options are currently limited. Most current treatment options primarily aim to slow the progression of the disease, rather than reversing the damage that has already occurred. Therefore, early detection, intervention, and ongoing monitoring and management of the disease are crucial for effective disease control.

Numerous previous studies have demonstrated that OCT is an effective tool for training CNNs to identify common retinal diseases such as dry AMD. Nonetheless, due to the complicated manifestations of nGA, implementing CNN models that classify the early stages of dry AMD is a significant challenge. The results of our experiments reveal that, among the evaluated CNN models, all models provide satisfactory classification performance for the normal and GA categories. However, nGA classification performance was generally inferior. This emphasizes the challenge of accurate classification of the nGA category and the need for novel approaches to improve the accuracy of classification.

We adapted normalization free ResNet50 as the backbone of the model, which is a variant of ResNet50. This variant omits normalization layers, which traditionally standardize the range of data features (34). The absence of normalization boosts computational efficiency and maintains a wider range of data features, thus enhancing the learning and performance capabilities in our specific image-based task of classifying dry AMD stages.

The proposed method's effectiveness can be attributed to its ability to combine image enhancement with hierarchical classification. An ablation study was conducted to compare the proposed method's performance with that of the baseline model and the baseline with image enhancement. The results demonstrated that hierarchical classification along with image enhancement significantly improves the classification performance for all categories, particularly nGA. The proposed method achieved the highest accuracy, sensitivity, specificity, f1 score, macro-f1 score, and kappa value for the three phases of dry AMD (drusen, nGA, and GA). The superiority of the proposed method suggests that it has the potential to be an effective and reliable tool for early detection and monitoring of AMD.

**Figure 6:**
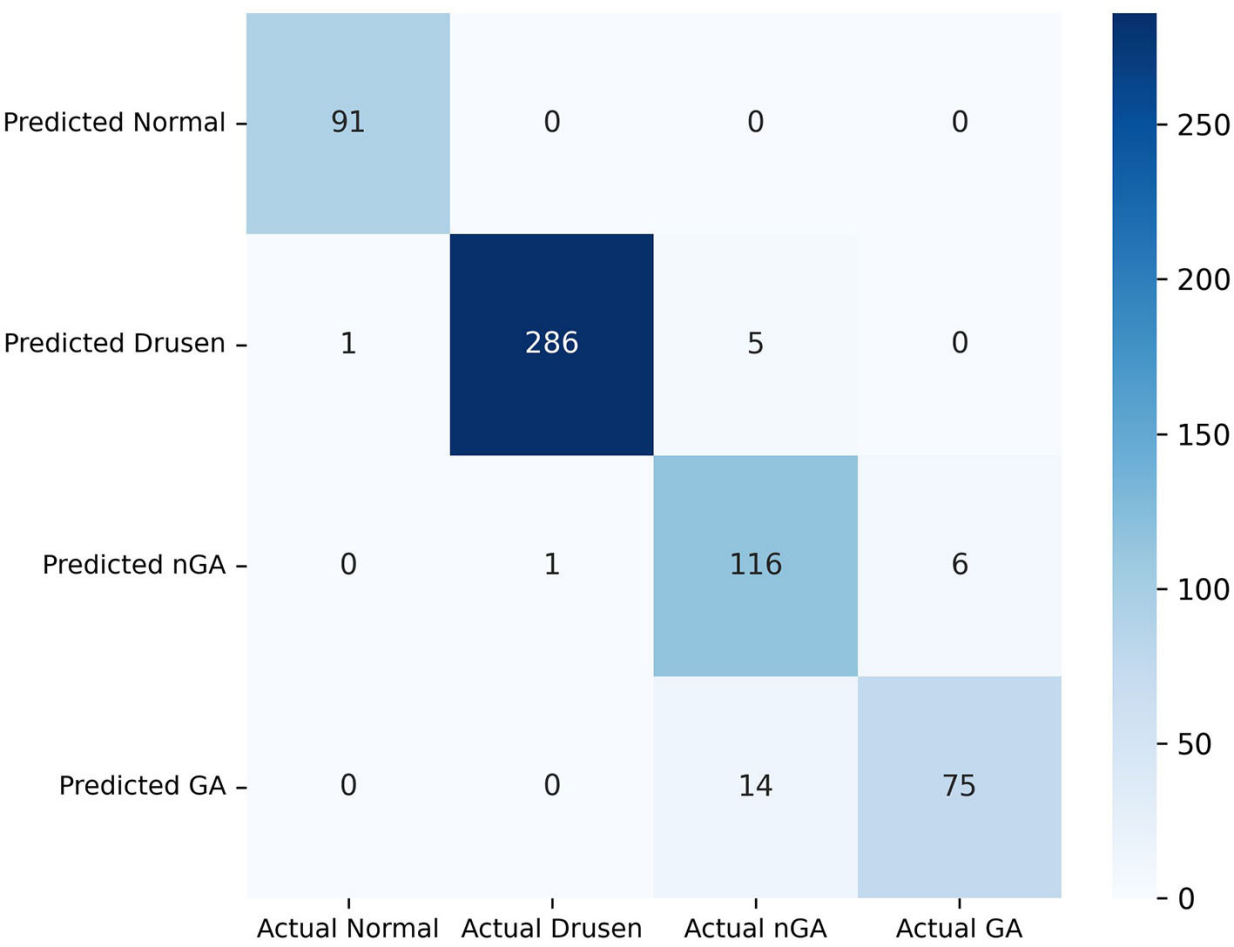
Confusion matrix of the proposed method validated on the Fold 1 dataset.

Our model exhibits substantial effectiveness in correctly predicting each phase of dry AMD, with high true positive values across all categories, as observed from the confusion matrix in Figure 6. A key observation is the high sensitivity in the identification of the drusen and GA phase. However, the matrix also highlights areas for potential improvement in the model, primarily in reducing false positives between the nGA and GA stages.

The image enhancement component improves the image quality, resulting in more accurate feature extraction and better CNN classification performance. The hierarchical classification component utilizes a two-step approach, in which the first CNN model classifies images into three categories, and then the second classification stage distinguishes between nGA and GA. This approach allows for accurate and reliable classification of nGA, which is a challenging category due to its similarity to drusen. These findings contribute to the development of a reliable and efficient automated diagnostic tool for early detection and monitoring of AMD, ultimately leading to improved patient outcomes. Further validation and testing of the proposed method on larger and more diverse datasets are necessary to confirm its generalizability and robustness.

**Figure 7:**
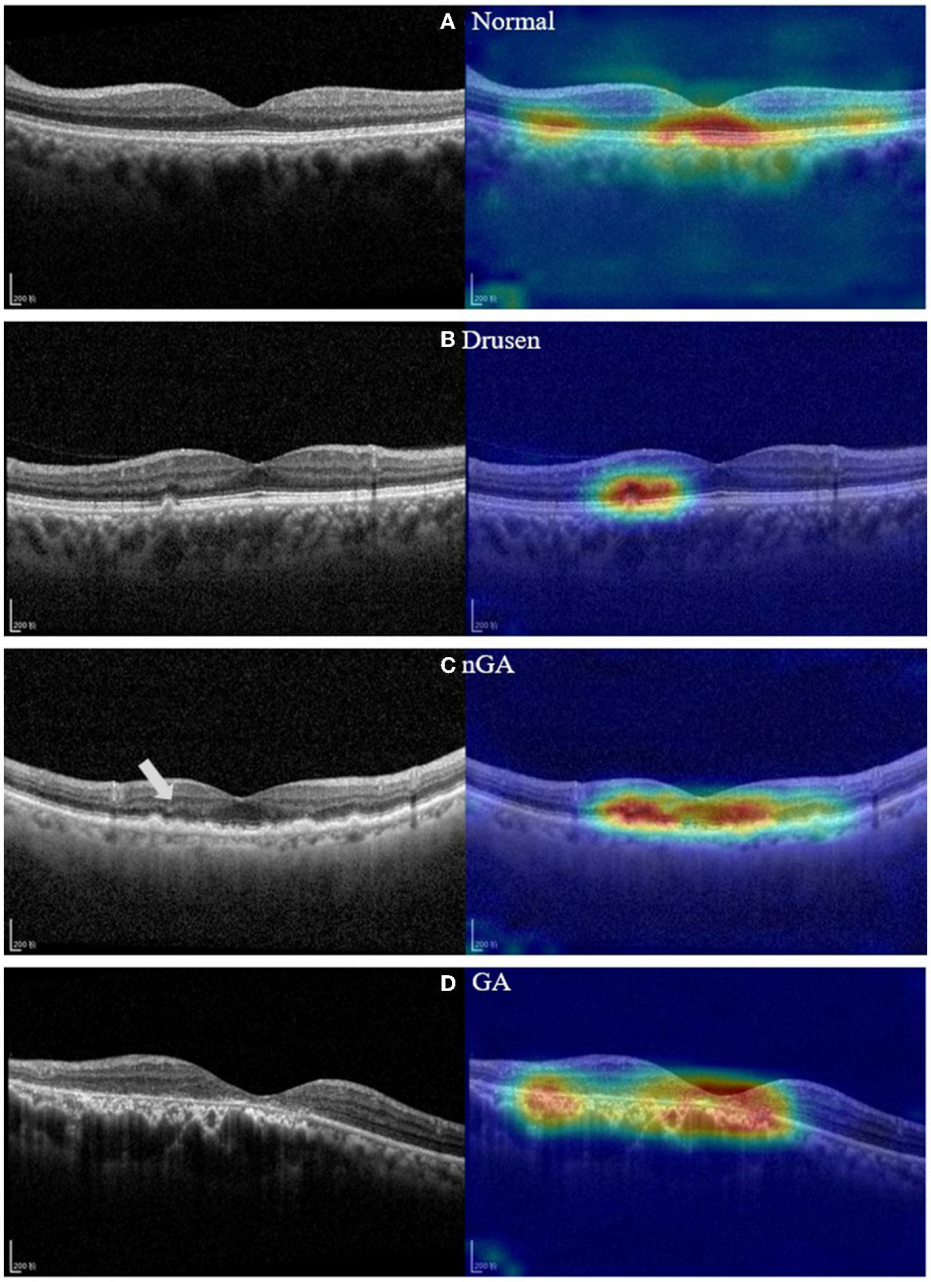
OCT images and feature heatmaps demonstrating example cases of normal, drusen, nGA and GA. **(A)** Example of OCT image of control group; **(B)** Example of a drusen regression detected by our model; **(C)** Example of nGA detected by our model. **(D)** Example of GA detected by our model. On the left side of the nGA example **(C)**, a discernible subsidence of OPL is marked.

Our model can precisely identify distinguishing characteristics within OCT images of different dry AMD phases as demonstrated by the heatmaps of Figure 7. In Figure 7B, the heatmap of our model focuses on the disruptions in the overlying retina layers, corresponding to the presence of drusen. This indicates that our model accurately identifies drusen features in this image. In Figure 7C, the model highlighted a relatively larger area in which OPL subsidence can be observed, as marked on the left side of the image. The heatmap of Figure 7D highlights regions where evidence of RPE thinning and depression of the outer retinal layers is present. These imaging features indicate the loss of retinal tissue and disruption of the normal retinal architecture, which are typically observed in GA.

### 4.1. Limitations

The present study has some limitations that could be addressed in future research. First, the study used the dataset from one center for evaluation. Further multi-center validation on larger and more diverse datasets is necessary to confirm the proposed method's robustness and generalizability. Second, the study evaluated four popular CNN models. It would be interesting to compare the proposed method's performance with state-of-the-art CNNs. Third, we did not perform exhausted fine-tuning on the image enhancement methods, which may have led to limited performance improvement solely through image enhancement. With further fine-tuning of the image enhancement stage, the image noise on the OCT images may be better eliminated, enabling the better identification of image characteristics for nGA. Future research should address these limitations and further validate and optimize the proposed method for practical implementation in clinical settings. Fourth, the phases of dry AMD was graded by a single clinician. While the clinician ensured a consistent evaluation standard across all OCT images, future work could benefit from double grading of OCT images by two or more experienced clinicians with consensus adjudication. Despite this limitation, our study has demonstrated the promising potential of our model for accurate classification of dry AMD stages.

Future work will expand upon this study by incorporating longitudinal OCT data from multiple follow-up visits to examine the progression of dry AMD from drusen to nGA and from nGA to GA. This will allow the investigation of the deep learning model's capability to identify and correctly predict the progression risk of dry AMD.

## 5. Conclusion

This study proposed and validated a novel two-step hierarchical CNN model with image enhancement for the classification of early-stage dry AMD, including the identification of nGA. The proposed method combines image enhancement with a CNN-based hierarchical model to improve the classification of early-stage dry AMD using OCT images. The results demonstrate the superior classification performance of the proposed method, particularly for the challenging nGA category. The proposed method's effectiveness could contribute to the development of a reliable and efficient automated diagnostic tool for early detection and monitoring of dry AMD. We believe that the proposed approach provides a valuable computer-assisted diagnostic tool for clinical diagnosis of dry AMD based on OCT. This could facilitate prompt and efficient interventions, leading to better management of the condition and patient outcome, and reducing the burden on ophthalmologists.

## Data availability statement

The data analyzed in this study is subject to the following licenses/restrictions: the data used in this study are not publicly available due to concerns about patient privacy. However, the code used to analyze the data and generate the results is available. The corresponding author can provide access to the code upon reasonable request. Any queries regarding the availability of materials should be addressed to the corresponding author. Requests to access these datasets should be directed to WD, daiweiwei@aierchina.com.

## Ethics statement

This study received ethics approval from the Ethics Committee of Shenyang Aier Excellence Hospital (approval number: 2021KJB002). The participants' confidentiality and anonymity were maintained throughout the study, and the research was conducted in accordance with the principles outlined in the Declaration of Helsinki. The study was conducted in compliance with all applicable ethical standards and regulations.

## Author contributions

MH and JX conducted the development of the models and drafted the manuscript. BW and DL contributed to the collection and labeling of the dataset used in this study. YC, ZY, and WD provided critical manuscript revisions and supervised the study. All authors contributed to the study concept and design, acquired and interpreted study data, and read and approved the final manuscript.
